# Biological Insights and Recent Advances in Plant Long Non-Coding RNA

**DOI:** 10.3390/ijms252211964

**Published:** 2024-11-07

**Authors:** Zhihao Zhao, Yaodong Yang, Amjad Iqbal, Qiufei Wu, Lixia Zhou

**Affiliations:** 1 National Key Laboratory for Tropical Crop Breeding, Chinese Academy of Tropical Agricultural Sciences, Haikou 571101, China; zzh5268@163.com (Z.Z.); yyang@catas.cn (Y.Y.); qfi_wu@catas.cn (Q.W.); 2 Hainan Key Laboratory of Tropical Oil Crops Biology/Coconut Research Institute, Chinese Academy of Tropical Agricultural Sciences, Wenchang 571339, China; amjadiqbal@awkum.edu.pk; 3 Industrial Development Department, Chinese Academy of Tropical Agricultural Sciences, Haikou 571101, China; 4 Department of Food Science & Technology, Abdul Wali Khan University Mardan, Khyber Pakhtunkhwa 23200, Pakistan

**Keywords:** plant, long non-coding RNA, regulation mechanism, biological functions

## Abstract

Long non-coding RNA (lncRNA) refers to an RNA molecule longer than 200 nucleotides (nt) that plays a significant role in regulating essential molecular and biological processes. It is commonly found in animals, plants, and viruses, and is characterized by features such as epigenetic markers, developmental stage-specific expression, and tissue-specific expression. Research has shown that lncRNA participates in anatomical processes like plant progression, while also playing a crucial role in plant disease resistance and adaptation mechanisms. In this review, we provide a concise overview of the formation mechanism, structural characteristics, and databases related to lncRNA in recent years. We primarily discuss the biological roles of lncRNA in plant progression as well as its involvement in response to biotic and abiotic stresses. Additionally, we examine the current challenges associated with lncRNA and explore its potential application in crop production and breeding. Studying plant lncRNAs is highly significant for multiple reasons: It reveals the regulatory mechanisms of plant growth and development, promotes agricultural production and food security, and drives research in plant genomics and epigenetics. Additionally, it facilitates ecological protection and biodiversity conservation.

## 1. Introduction

According to the classical central dogma, the general process of gene expression is to generate RNA through DNA transcription, then translate it into protein, and finally realize the biological function [1]. Among these, RNA is considered to be the only messenger carrying information in the process. The rapid advancement of high-throughput transcriptome sequencing and expression profiling technologies, combined with the ongoing collection of transcriptome data from both animals and plants, has significantly enhanced researchers’ ability to investigate the molecular mechanisms of genes. This progress has particularly focused on areas such as biological growth and development as well as the transcriptional regulation of stress responses [2]. Extensive genomic sequencing data demonstrate that a significant portion of DNA sequences in eukaryotic genomes is transcribed into RNA; however, fewer than 2% of these sequences correspond to protein-coding genes. Moreover, over 90% of the remaining genomic sequences are transcribed into non-coding RNA, which does not code for proteins. This category includes long non-coding RNAs (lncRNAs), transfer RNAs (tRNAs), micro RNAs (miRNAs), ribosomal RNAs (rRNAs), small nuclear RNAs (snRNAs), small interfering RNAs (siRNAs), and circular RNAs [3,4,5]. LncRNAs are a type of non-coding RNA that are longer than 200 nucleotides (nt). They can be categorized into various types, including intronic, intergenic, exonic, sense, and anti-sense categories. LncRNAs are located within or near protein-coding genes. They also originate from chromosomal regions with low gene density, such as pericentromeres and telomeres. LncRNAs play a crucial task in regulating numerous molecular processes [6]. These include mRNA stability, transcription, splicing, interactions between chromosomes, chromatin structure, nucleosome remodeling, post-translational modifications, and the organization of nucleosomes. The majority of lncRNAs are produced by RNA polymerase II, transcribing from either the sense or anti-sense strands [7]; however, a small subset is generated by alternative RNA polymerases, such as Pol IV and Pol V [8]. LncRNAs typically possess a 5′ cap, poly-A tail, and fewer introns. They are characterized by low abundance, tissue specificity, and low sequence conservation among different species. LncRNAs can interact with DNA, RNA, and proteins to perform their tasks. They can function in either a cis or trans manner and are found in both the nucleus and the cytoplasm [9,10,11]. Most lncRNAs are polyadenylated; however, in yeast and mammals, there are also numerous non-polyadenylated lncRNAs present [12]. Nonetheless, several important functional non-polyadenylated lncRNAs have been extensively researched. Recent studies in *Arabidopsis* revealed that abiotic stress triggers the production of hundreds of these non-polyadenylated lncRNAs [13].

Nowadays, both the identification and functional research of plant lncRNAs are receiving increasing attention in the field of scientific research. Numerous lncRNAs with key regulatory functions have been discovered and validated [14]. Genome-wide array analysis and RNA-seq results demonstrated a substantial presence of lncRNAs in plants, which regulate gene expression via mechanisms such as DNA methylation, histone methylation, and transcriptional interference mechanisms. LncRNAs are crucial regulators in various biological processes, including male sterility, flowering, transposon activity, and responses to both biotic and abiotic stress [15]. A plant lncRNA database has been constructed with over one million lncRNAs from more than 80 plant species [16]. This paper reviews the structural features and classification of plant lncRNAs, the research approaches used in studying lncRNAs, and the challenges encountered in current investigations. It aims to provide a theoretical foundation and new perspectives for future lncRNA research.

LncRNAs perform important regulatory roles in plants, impacting processes such as cell growth, proliferation, differentiation, and apoptosis. Studying the functions of lncRNAs enhances our understanding of the molecular mechanisms behind plant growth and development, offering a theoretical foundation for crop improvement and breeding. Understanding how lncRNAs function in plants can assist in developing crop varieties with greater stress resistance and higher yields. Regulating lncRNA expression can improve plant tolerance to challenges like drought and salinity/alkalinity, enhancing crop yield, adaptability, and food security. LncRNAs are also key performers in epigenetic regulation. They can recruit chromatin remodeling and modification complexes, change the DNA/RNA methylation status as well as the chromosome structure and modification status, and then control the expression of related genes. Investigating lncRNAs is helpful for an in-depth understanding of plant genomics and epigenetics and promoting the development of related fields.

Plants are an important part of the ecosystem; accordingly, exploring the functions of lncRNAs in understanding how plants adapt to their environment—thereby supporting the ecological environment and biodiversity—is crucial. For example, by regulating the expression of lncRNAs, the tolerance of plants to environmental changes can be enhanced, protecting the stability of the ecosystem. In fact, investigating the biological functions of non-coding RNAs in plants offers multiple benefits: It not only reveals the molecular mechanisms of plant growth and development but also supports agricultural production and food security. Additionally, it drives genomics and epigenetics research and contributes to ecological protection and biodiversity conservation.

## 2. LncRNA Genomic Regulation and Plant Biology

### 2.1. Generation and Classification of LncRNAs

According to the classical central dogma, RNA is frequently viewed as a link between DNA and protein. The research conducted during the Human Genome Project revealed that only 2–5% of genes in the human genome are capable of encoding proteins, while the majority consists of non-coding RNA (ncRNA) [17]. NcRNA widely exists in many organisms. According to its functions, ncRNA can be divided into small nucleolar RNA (snoRNA), snRNA, rRNA, and tRNA [18]. According to the different lengths of ncRNA molecular chain [19], it can be divided into lncRNA and short non-coding RNA (sncRNA). LncRNA is a type of RNA that is longer than 200 nucleotides (nt) and is transcribed by RNA polymerase II but it does not serve a protein-coding function. An open reading frame (ORF) is typically short, usually containing fewer than 100 amino acids (aa), and its expression level in vivo is quite low. Additionally, lncRNA and the mRNA protein-coding genes have distinct subcellular localizations within the nucleus [20,21]. Most lncRNAs mainly exist in the chromatin of the nucleus and in one or two subcellular chambers. LncRNAs have a relatively intricate secondary structure and are found in both the nucleus and cytoplasm, exhibiting spatiotemporal expression specificity [22].

LncRNAs can be generated through five mechanisms [23]: (1) The insertion of a reading frame occurs due to protein-coding genes, with the newly inserted reading frame combining with the existing coding sequence to form a new functional lncRNA. (2) After chromosome recombination, two non-transcribed, distant sequence regions merge to form an lncRNA that contains multiple exons. (3) A non-coding gene is copied through retro-transposition, resulting in either a functional non-coding reverse gene or a nonfunctional reverse gene. (4) Two consecutive repeat events form adjacent repeat sequences within the ncRNA to produce lncRNA. (5) The insertion of a transposition factor yields functional lncRNA.

To date, lncRNAs are categorized according to their intrinsic characteristics, which include transcription length, relationship with protein-coding genes, DNA elements, and the presence of other known functional repeats [24]. These characteristics influence the DNA sequence, biochemical pathways, stability, structural protection, physiological conditions, functions, and targeting mechanisms within the biological genome (Table 1).

### 2.2. Structure and Characteristics of Long Non-Coding RNA

The primary characteristics of lncRNA include a length greater than 200 nt and the absence of protein-coding capability [30]. LncRNAs generally possess a limited number of exons and introns and lack an ORF, start codon, and stop codon, which might be partially connected to their low prevalence and fragmented assembly. The majority of lncRNAs are transcribed by RNA polymerase II and possess a 5′ cap along with a 3′ polyadenylate tail. The transcripts of lncRNA are shorter than those of mRNA [31].

As a molecule with multiple biological functions, lncRNA is instrumental in embryo development, tumor formation, material metabolism, protein localization, etc., but research into its mechanisms is very limited [20]. Defining the structure of RNA is the first step in studying its functions and mechanisms. One of the most important characteristics of lncRNA is its complex secondary and tertiary structures, which are closely related to the biological function. In contrast to proteins, the primary structure of lncRNA exhibits low conservation and has therefore often been regarded as transcriptional noise [32]. However, its secondary and tertiary structures are highly conserved. The secondary structure of lncRNA comprises helices, loops, and single strands. The tertiary structure is stabilized by the coaxial stacking of kissing loops and spiral regions [33].

### 2.3. Multi-Omics for LncRNA Functional Annotation

Genomic sequencing techniques, such as RNA-seq in next-generation sequencing (NGS), enable the identification of fundamental gene structures of lncRNAs, including transcript boundaries and exon–intron structures. For instance, compared to coding genes, lncRNAs typically contain fewer exons and have shorter exon lengths. By sequencing and analyzing numerous lncRNA transcripts, their structural characteristics can be summarized, providing a basis for further functional research. Bioinformatics tools are employed at the genomic level to analyze the promoter regions of lncRNAs to predict possible transcription factor binding sites (TFBS) [34]. For example, by analyzing chromatin immunoprecipitation sequencing (ChIP-Seq) data, researchers can identify the transcription factors that interact with the promoter regions of lncRNAs, enabling insights into the transcriptional regulatory mechanisms governing lncRNAs. If a particular transcription factor binding site is found in the promoter region of an lncRNA, and that transcription factor is associated with a specific biological process, it suggests that the lncRNA may play a role in regulating that biological process.

RNA-seq technology can be used to analyze the expression of lncRNAs in different tissues and developmental stages. By comparing the lncRNA expression profiles from different tissues or developmental stages, lncRNAs that are highly expressed in specific tissues or developmental periods can be screened out. These lncRNAs are likely to have significant roles in the development of the corresponding tissues or in their physiological functions [20]. Co-expression analysis between lncRNAs and coding genes allows for the identification of groups of coding genes that share similar expression patterns with lncRNAs. For example, by calculating the expression correlations between lncRNAs and all coding genes across various samples, researchers can identify coding genes that are either positively or negatively correlated with lncRNAs [35]. If an lncRNA shows co-expression with a set of coding genes associated with cell metabolism, it may indicate that this lncRNA contributes to the regulation of cell metabolic processes. This co-expression occurs because genes that are co-expressed frequently participate in similar or interconnected biological processes. The DNA methylation status of lncRNA gene regions can be assessed using techniques such as whole-genome methylation sequencing (WGBS) or reduced-representation bisulfite sequencing (RRBS). Studies have shown that the expression of lncRNAs can be regulated by DNA methylation. Certain lncRNAs are capable of interacting with DNA methyltransferases (DNMTs) to affect the DNA methylation status of genes. For example, lncRNA-HOTAIR can guide DNMTs to specific gene loci, resulting in elevated gene methylation that subsequently suppresses gene expression [36]. Investigating the interactions between lncRNAs and enzymes associated with DNA methylation can provide valuable insights into the roles of lncRNAs in epigenetic regulation.

ChIP-seq technology can be used to study the relationship between histone modifications (such as H3K4me3, H3K27me3, etc.) and lncRNA transcription. For instance, the presence of H3K4me3 modification in the promoter region of an lncRNA gene typically signifies an active transcriptional state, whereas the H3K27me3 modification is related to gene silencing [37]. Analyzing the patterns of histone modification in lncRNA gene regions can provide insights into the transcriptional regulatory mechanisms. Certain lncRNAs have the ability to interact with histone-modifying enzymes, affecting chromatin structure and gene expression. For example, lncRNA-Xist is crucial for X-chromosome inactivation; it binds to histone-modifying enzymes, such as polycomb repressive complex 2 (PRC2), which results in increased histone H3K27me3 modification on the X-chromosome, effectively silencing it. By investigating the interactions between lncRNAs and histone-modifying enzymes, the epigenetic mechanisms of lncRNAs in gene expression regulation can be revealed [38]. Through RNA-protein immunoprecipitation (RIP) combined with mass spectrometry (MS) technology, proteins that interact with lncRNAs can be identified. For example, cell lysates are immunoprecipitated with antibodies that specifically recognize the target lncRNA, and then the precipitated proteins are analyzed by mass spectrometry. If an lncRNA is found to interact with a protein with a known function, such as a transcription factor or a key protein in a signaling pathway, then it can be speculated that the lncRNA may play a role by affecting the function of this protein [39]. The influence of lncRNA protein interactions on protein functions, such as protein stability, subcellular localization, or activity, is being investigated. For instance, certain lncRNAs can bind to proteins, inhibiting their degradation by proteases and thus enhancing protein stability. Additionally, lncRNAs can guide the proteins bound to them to specific subcellular locations, affecting the function of proteins in cells. These studies provide deeper insights into the mechanisms by which lncRNAs operate within cells.

### 2.4. Functional Roles of LncRNAs in the Regulation of Gene Expression

LncRNAs are typically found in the nucleus of eukaryotic cells, where they perform anatomical functions through various mechanisms. Unlike post-transcriptional regulation by miRNAs, which bind to the 3′ untranslated region (3′ UTR) of the target gene’s mRNA, lncRNAs can regulate gene transcription through many mechanisms. Researchers have proposed the following three modes of action:

(1) Bait: LncRNAs can function as decoys to inhibit the binding of regulatory proteins to DNA. For instance, lncRNA Gas5 features a hairpin sequence that resembles the DNA binding domain of the glucocorticoid receptor. Starvation can induce the expression of Gas5, which then functions as a decoy to facilitate the release of the glucocorticoid receptor from its DNA binding site, thereby inhibiting the transcription of genes associated with metabolism [40]. Similarly, lncRNA PANDA can prevent apoptosis mediated by p53 by binding with the transcription factor NFYA through a similar mechanism [41];
(2) Scaffolding or bridging: LncRNAs serve as regulators that guide two or more proteins into specific networks. As a scaffold, telomerase RNA TERC participates in the assembly of the telomerase complex [42]. Similarly, HOTAIR operates as a structural framework by combining the PRC2 and LSD1/CoREST complexes within a particular domain;
(3) Guide: The binding of specific protein complexes to specific DNA regions requires the guidance of lncRNAs. Long non-coding RNAs, which play a guiding role, have two main functions—binding specific proteins and selectively acting on certain regions of the genome by some mechanism. Additionally, lncRNAs with a guiding function can exert their biological roles through either *cis* or *trans* action [43,44].

### 2.5. The Databases of Plant Long Non-Coding RNA

Improvements in transcriptome arrays and deep sequencing technologies have led to the swift accumulation of extensive datasets of lncRNAs such as *Arabidopsis thaliana* [45], *Triticum aestivum* [46,47], *Oryza sativa* [48,49], *Zea mays* [50,51], *Medicago sativa* [52], and *Solanum lycopersicum* [53]. Recently, lncRNA transcripts and the associated information have been compiled in databases specifically focused on lncRNA research. In this context, we provide an overview of the databases related to plant lncRNAs, with details presented in Table 2.

The CANTATA database (http://yeti.amu.edu.pl/CANTATA/ accessed on 12 September 2024) provides information on lncRNAs from a wide range of plant species. It primarily relies on software predictions to identify lncRNAs. At present, it holds 239,631 lncRNAs from 39 species, making it the largest plant lncRNA database available [54,55].

NONCODE (http://www.noncode.org/ accessed on 12 September 2024) operates as an accessible interactive database that is designed to provide the most extensive collection and detailed annotation of non-coding RNAs, with a particular focus on long non-coding RNAs [59]. Now, there are 17 species in NONCODE, and the number of lncRNA in the database has increased from 527,336 to 548,640. Taking everything into account, NONCODE aims to offer the most comprehensive collection and annotation of non-coding RNA. It provides not only basic details like location, length, sequence, exon number, and strand of lncRNAs but also more advanced information, including the conservation info, exosome expression profile, expression profile, disease relation, and predicted function.

The Green Non-Coding Database (GreeNC) (http://greenc.sequentiabiotech.com/wiki2/Main_Page accessed on 5 November 2024) is a repository dedicated to the annotation of lncRNAs in algae and plants [60]. This database uses a transcript filtering method instead of a machine learning approach, where transcripts must fulfill the criteria of a typical lncRNA in order to be classified as potential lncRNAs. For a transcript to be classified as an lncRNA in the GreeNC database, it must be longer than 200 nt, contain an ORF smaller than 120 aa, have no match in the SwissProt database, and be identified as non-coding by the Coding Potential Calculator. The GreeNC database offers details on genomic coordinates, folding energy, coding potential, and sequence for all identified lncRNAs.

The plant long non-coding RNA database (PlncDB) (http://bis.zju.edu.cn/PlncRNADB/index.php accessed on 21 September 2024) is a data repository focusing on the analysis of lncRNA in plants [61]. This database contains lncRNA information on *Arabidopsis*, *Populus trichocarpa*, and *Zea mays*. The RNA-seq sequencing reads of species are collected from the GEO database and the transcripts are assembled. Once lncRNAs are identified, their interactions with proteins are predicted using the online catRAPID service. This database offers four aspects of functions related to lncRNAs: (1) Genomic information for a significant number of lncRNAs acquired from multiple sources. (2) A web-based genome browser for exploring plant lncRNAs leveraging a platform aligned with the UCSC Genome Browser. (3) Integration of transcriptome databases obtained from a variety of samples, including transition phases, distinct tissues, mutants, and stress therapies. (4) A collection of datasets related to epigenetic modifications and small RNA is available. PLncDB offers an extensive genomic overview of *Arabidopsis* lncRNAs serving the plant science consortium. This database is routinely updated with new plant genomes as they become available, significantly aiding future research on plant lncRNAs.

JustRNA, a resource platform for the expression profiling analysis of plant lncRNAs, currently contains annotations for 1,088,565 lncRNAs from 80 different plant species [16]. Additionally, it includes 3692 RNA-seq samples from 825 conditions across six model plants. Functional network reconstruction provides deep insights into the regulatory roles of lncRNAs. Genome-wide association studies and microRNA target predictions can be used to map potential interactions with neighboring genes and microRNAs, respectively. Subsequent co-expression analysis can be employed to enhance confidence in gene-to-gene interactions. By integrating chromatin immunoprecipitation sequencing data for transcription factors and histone modifications into JustRNA, the platform identifies transcriptional regulation of lncRNAs in several plants. The JustRNA platform offers researchers valuable insights into the regulatory mechanisms of plant lncRNAs.

## 3. Expression of LncRNAs in Plants and Their Biological Functions

**Figure 1:**
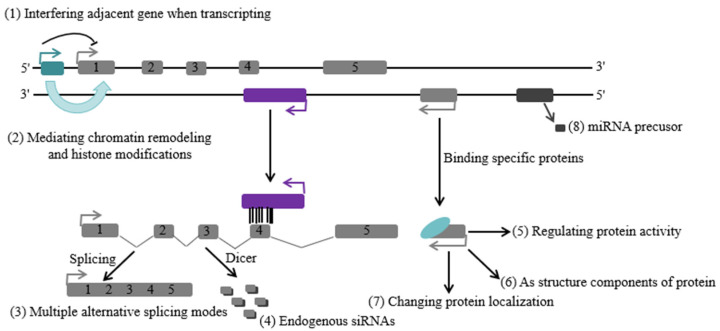
The mechanisms of lncRNAs.

LncRNAs are involved in numerous biological activities of organisms, regulating gene expression through multiple modes of action, primarily manifested in the regulation of networks covering transcriptional, post-transcriptional, translational, and epigenetic levels (Figure 1) [60].

### 3.1. Functions of LncRNAs in Plant Progression

LncRNAs perform a regulatory role in auxin transport and development signal output. In *Arabidopsis*, the auxin-regulated promoter loop (APOLO) gene is situated 5148 bp prior to the PID gene and is expressed by RNA polymerases II and V as a result of auxin. The lncRNA produced by transcription can dynamically regulate the cyclization process of the PID promoter, thereby affecting the expression mode of the *PID* gene [62]. The circadian rhythm of gene expression results from a series of biological processes. FLORE, identified as the natural anti-sense transcript of CDF5, is among the *Arabidopsis* lncRNAs and is associated with the regulation of the biological clock. The circadian regulation of FLORE was confirmed using real-time quantitative RT-PCR. Finally, a new module of circadian regulation, the natural anti-sense transcription pair of CDF5/FLORE, was proposed, which has the characteristics of conservative mutual inhibition and unique trans action, can precisely regulate the circadian rhythm change, and promote the plant to start flowering [63]. Conversely, lncRNAs can act as sources of miRNAs or influence the accumulation and activity levels of miRNAs at both transcriptional and post-transcriptional stages [64]. One of the primary mechanisms by which lncRNAs and miRNAs operate is through the sequestration of miRNAs, which lowers their expression levels and reduces mRNA repression, ultimately affecting the translation rate and stability of downstream target genes. For instance, IPS1 and At4 can competitively interact with miR399, resulting in elevated expression levels of PHO2. Both miR399 and PHO2 are essential for regulating phosphate homeostasis in *Arabidopsis thaliana* [65]. This precise regulation of miRNA activity by endogenous non-cleavable lncRNA targets is referred to as targeting.

To investigate the significance of lncRNA1459 in the ripening of *Solanum lycopersicum* fruit, the lncRNA1459 mutant was created using CRISPR/Cas9 gene editing technology. The lncRNA1459 mutant exhibited a marked delay in ripening compared to the wild type, along with a substantial decrease in both ethylene production and lycopene accumulation. In the knockout mutants of lncRNA1459, numerous genes associated with aging of *S. lycopersicum* fruit and the expression of lncRNAs also exhibited significant changes. In the analysis of mutant lines, a positive correlation was found between the expression of lncRNA1459 and 81 differentially expressed lncRNAs, while 31 differentially expressed lncRNAs showed a negative correlation. The results suggested that lncRNA1459 has a regulatory task in the transcription of genes and lncRNAs involved in the ripening of *S. lycopersicum* fruit [66].

In *Arabidopsis*, nuclear speckle RNA binding proteins (NSRs) perform a task in regulating multiple splicing processes. They have the ability to bind to mRNA and lncRNAs like ENOD40 and lnc351. Together, these lncRNAs are referred to as ASCO-RNA (alternative splicing competitor RNA). ASCO-RNA competes with mRNA for binding to NSRs, which disrupts the splicing of downstream auxin response genes by NSR proteins. This interference ultimately affects the growth of lateral roots [67].

### 3.2. LncRNAs Participate in Plant Reproductive Growth

Plant lncRNAs are crucial for transcription, post-transcription, and epigenetics. Their influence on reproductive development includes regulating flowering, determining the differentiation of male and female plants, and impacting pollen development. Gaining insight into the impact of lncRNA in plant reproductive development is essential for understanding pollen sterility resulting from disruptions in communication between organelles and the nuclear genome [68]. Long day-specific male fertility-associated RNA (LDMAR) is a 1236-nucleotide lncRNA that plays a role in regulating reproductive development in *Oryza sativa*. Under sustained light conditions, the expression of LDMAR maintained the normal development of *O. sativa* pollen. Due to the change in the LDMAR’s secondary structure caused by a single base mutation, the methylation degree in the LDMAR’s promoter region was increased, the expression of LDMAR was decreased, the programmed death of pollen was caused, and this eventually led to a photosensitive male sterile line [69,70].

Research focused on *Arabidopsis* has demonstrated that lncRNAs regulate the initiation of flowering by influencing the expression of FLOWERING LOCUS C (FLC), a gene that encodes a MADS-box transcription factor. FLC inhibits the expression of downstream genes necessary for flowering, thereby negatively regulating the flowering process in a dose-dependent manner. FLC is involved in the vernalization pathway, which adjusts the flowering time in response to extended periods of low temperature, as well as in the autonomous pathway, which regulates flowering time independently of environmental influences [71]. The involvement of the lncRNA COLDAIR in its interactions with Polycomb Repressive Complex 2 (PRC2) and the FLC during the vernalization process in *A. thaliana* was highlighted by Kim et al. [72]. The repression of FLC is facilitated by the enhanced recruitment of PRC2, leading to the trimethylation of Histone H3 at Lysine 27 (H3K27me3) within the FLC chromatin. The interaction of COLDAIR with chromatin is specific to the FLC locus, and the central region of the COLDAIR transcript is essential for this association. A modular region in COLDAIR is accountable for the alignment with PRC2 in vitro, and mutations within this motif that diminished COLDAIR with PRC2 led to insensitivity to vernalization. The vernalization insensitivity resulting from the mutant COLDAIR was restored through the ectopic expression of the wild type COLDAIR. A total of 6510 lncRNAs were identified in *Arabidopsis* under both normal and stress conditions using high-depth strand-specific RNA sequencing (RNA-seq) in a study conducted by Zhao et al. [73]. Their research revealed that the expression of natural anti-sense transcripts (NATs), which are transcribed in the reverse direction of protein-coding genes, often shows a positive correlation with and is essential for the expression of their corresponding sense genes. NATs play an essential role in regulating sense gene expression. Additionally, they identified a specific NAT lncRNA, MAS, which enhances the transcription of its corresponding sense gene *MAF4*. MAS achieves this by interacting with and recruiting WDR5a, a key component of the COMPASS-like complexes, and recruiting it to *MAF4*. This interaction ultimately influences flowering time [73]. *O. sativa* lncRNAs exhibited distinct expression patterns depending on specific tissues and developmental stages. Certain lncRNAs acted as endogenous competitive RNAs (ceRNAs), engaging in target mimicry by sequestering miR160 or miR164. Essentially, one lncRNA, XLOC_057324, was shown to be involved in regulating panicle development and fertility [74]. A total of 152, 233, and 197 differentially expressed lncRNAs were identified across the three stages of ovule development in *O. sativa* [75]. The functional analysis of the target genes associated with differentially expressed lncRNAs revealed that numerous lncRNAs are involved in various pathways, namely hormone regulation, signal transduction, and cellular metabolism. In addition, several differentially expressed lncRNAs served as the precursors to specific miRNAs, which performed roles in ovule and female gametophyte development. It was also discovered that lncRNAs can function as decoys, competing with mRNAs for miRNAs binding to regulate the normal expression of genes involved in ovule and female gametophyte development [75].

### 3.3. NA Participates in Abiotic Stress Responsive Regulation

Plant productivity can be severely affected by major abiotic stresses, mainly drought, cold, and salt. To cope with these stresses, plants modify their metabolism, physiology, and development, which can help alleviate some of the adverse effects on their growth and reproduction [76]. Previous studies on the regulation of stress-related genes have primarily concentrated on genes that encode proteins. In recent decades, non-coding RNAs that do not code for proteins have been increasingly recognized for their significance. Growing evidence indicates that non-coding RNAs, particularly lncRNAs, perform a crucial role in modulating responses to various abiotic stresses [77,78].

#### 3.3.1. Drought Stress

It was found that At5nc056820 is an lncRNA that responds to drought stress and abscisic acid treatment in *A. thaliana*, with its expression exhibiting significant changes, potentially indicating its role in drought response [79]. Transgenic lines A-3, A-7, and A-8 were developed by introducing At5nc056820 into *A. thaliana*. The drought resistance tests revealed that these transgenic lines exhibited superior performance compared to the wild type, particularly showing a 2.2 to 2.5-fold increase in free proline content compared to the wild type. These findings suggest that At5nc056820 can enhance the drought resistance of *A. thaliana* to a certain degree [80]. A novel drought-induced lncRNA (a drought-induced RNA, DRIR) was identified, which enhanced the tolerance of *A. thaliana* to both drought and salt stress. Recognized as a novel positive regulator of plant responses to drought and salt stress, this RNA was activated following treatment with drought, salt stress, and abscisic acid (ABA), resulting in a significant increase in its expression level [81]. Transcriptome analysis revealed that DRIR modulates genes associated with ABA signaling, water transport, and various mechanisms for alleviating stress [76]. A substantial number of lncRNAs associated with the drought memory response was identified through whole-transcript strand-specific RNA sequencing (ssRNA-seq). This research illustrated that *O. sativa* can develop stress memory when subjected to suitable levels of water deficiency. It also indicated that lncRNA, DNA methylation, and endogenous phytohormones (particularly ABA) perform tasks in short-term drought memory in *O. sativa*. These factors may function as memory components that activate drought-related transcripts in pathways, chiefly photosynthesis and proline biosynthesis, enabling the plant to better respond to the subsequent stressors [82].

A significant number of lncRNAs associated with drought stress in *Gossypium herbaceum* were identified using a reproducible RNA sequencing and bioinformatics approach. Particularly, the lncRNAs XLOC_063105 and XLOC_115463 perform tasks in regulating two adjacent coding genes, CotAD_37096 and CotAD_12502, respectively [83]. AtDANA2, derived from *Arabidopsis*, serves as a positive regulator of the drought response and collaborates with the transcriptional activator ERF84 to regulate the expression of JMJ29 in response to drought conditions [84]. Analysis of the characteristics and the expression patterns of lncRNAs during drought stress and subsequent rewatering treatment revealed that lncRNAs may perform a significant function in regulating plant hormone pathways in response to drought conditions.

#### 3.3.2. Cold Stress

A total of 12,462 lncRNAs were identified in cold-stressed wild *Musa paradisiaca* using RNA sequencing. By elucidating the genome-wide gene expression patterns and alterations in lncRNA levels using high-throughput RNA sequencing, the regulatory pathways involved in cold responses in wild *M. paradisiaca* were clarified. Identifying lncRNAs that respond to cold stress enhances the understanding of cold resistance in *M. paradisiaca* species and offers valuable insights for breeding programs aimed at improving cold tolerance [85]. The expression dynamics of lncRNAs in *Vitis vinifera* subjected to cold stress were investigated by high-throughput sequencing. The analysis showed that, in grapevines subjected to cold treatment, there was a notable increase in the expression of 203 identified lncRNAs, while the expression of 144 known lncRNAs was significantly decreased. Furthermore, the same study identified 2088 previously uncharacterized lncRNA transcripts, of which 284 novel lncRNAs were found to be substantially up-regulated and 182 considerably down-regulated in *V. vinifera* exposed to cold treatment. Additionally, 242 differentially expressed lncRNAs in *V. vinifera* were predicted to have a cis-regulatory relationship with 326 protein-coding genes [86]. Since miR398 has been shown to react to various stress conditions [83], Lu et al. [87] investigated how miR398 interacts with lncRNA in the context of cold resistance in winter *Triticum aestivum*. The findings indicated that tae-miR398 performs a task in enhancing low-temperature tolerance by suppressing its target, CSD1. The expression of CSD1 was influenced indirectly by lncRNA through competitive binding with miR398, subsequently impacting the cold resistance of Dn1. This regulation by miR398 initiated a feedback loop that was essential for cold stress tolerance in *T. aestivum*.

#### 3.3.3. Salt Stress

Soil salinization has emerged as a significant challenge in global land management. The presence of salt in the soil negatively impacts plant processes, such as photosynthesis and seed germination. When salt ion concentrations rise beyond a certain threshold, they can trigger toxicity, osmotic stress, and oxidation stress, resulting in nutrient deficiencies, stunted growth, and ultimately, plant death [88].

A total of 23,324 lncRNAs were discovered in *Medicago truncatula* under salt stress conditions. Specifically, 7361 lncRNAs were identified in leaves subjected to salt stress, while 7874 were found in stressed root samples. Under salt stress conditions, the roots exhibited a greater number of differentially expressed lncRNAs compared to the leaves. However, when exposed to osmotic stress, leaves had a significantly higher number of unique lncRNAs than the roots [89]. Through deep RNA sequencing, researchers identified lncRNAs in Lluteño *Zea mays* in response to the combined stresses of salinity and excess boron. They reported 48,345 distinct lncRNAs in the salt-tolerant Chilean *Z. mays* variety ‘Lluteño’, with 41.9% of them being unique to the transcriptome of *Z. mays*. A set of 848 stress-responsive potential trans natural anti-sense transcripts (trans-NAT) lncRNAs were identified and confirmed. These lncRNAs appear to regulate genes involved in stress responses, transcriptional control, reactions to abiotic stimuli, and the nicotianamine metabolic pathway [90]. Using whole-transcript strand-specific RNA sequencing, researchers analyzed cotton seedlings at the three-leaf stage when exposed to salt stress and identified 1117 distinct lncRNAs. Among them, lnc_388 was suggested to regulate Gh_A09G1182, while lnc_973 and lnc_253 were predicted to act as target mimics for ghr-miR399 and ghr-156e under salt stress conditions [91].

Researchers conducted RNA sequencing of three different RNA libraries (poly(A)+, poly(A)-, and nuclear RNAs) in *O. sativa* plants subjected to four abiotic stress conditions: salt, heat, cold, and drought. Transcriptome sequencing revealed the discovery of over 7000 lncRNAs, with almost half of them being newly identified. Conspicuously, a large portion of the approximately 500 poly (A) lncRNAs exhibited differential expression under stress conditions, with a significant reduction in their expression levels. Additionally, numerous lncRNAs with down-regulated polyadenylation (DPA) appeared to be highly conserved, exhibit notable nuclear retention, and are co-expressed with protein-coding genes involved in stress responses. Strikingly, these DPA lncRNAs showed significant enrichment within quantitative trait loci (QTLs) associated with stress tolerance and developmental traits [48].

### 3.4. LncRNA Participates in Biotic Stresses

Plants experience the effects of not only abiotic stress but also a range of biological stresses throughout their growth and development phases [11]. They frequently encounter disturbances caused by viruses, fungi, bacteria, and pests [92]. Plants have developed a range of effective mechanisms for disease suppression and defense to reduce the damage they face. Their reaction to pathogen invasion is contingent upon the recognition of the pathogen at the cellular level. In the last few years, many lncRNAs have been found to perform a role in biotic stresses. Acting as targets for microRNAs, lncRNAs can suppress the activity of specific miRNAs and activate intricate defense signaling networks, facilitating the reprogramming of transcription [12].

Long intergenic ncRNA (lincRNA) 1, a form of ncRNA, is commonly found in cells and performs a meaningful role in multiple biological regulatory mechanisms. LincRNAs are typically shorter than lncRNAs and are mainly derived from the disordered expression of genes during tissue development. They can indirectly influence the expression of corresponding genes by affecting the processing, transport, or translation of RNA molecules. In contrast, lncRNA mainly arises from the normal expression of genes and primarily functions to promote, inhibit, or interfere with gene expression. A total of 1113 lincRNAs were identified as being expressed across all 12 chromosomes of *Solanum tuberosum*. Of these, 559 lincRNAs were found to be responsive to the pathogen *Pectobacterium carotovorum* subsp. Brasiliense. These findings indicate that lincRNAs may execute a responsibility in the defense mechanisms of *S. tuberosum* [93]. Through comparative transcriptome analysis of *S. tuberosum* resistant and susceptible to *Phytophthora infestans*, differentially expressed genes (DEGs) and long non-coding RNAs (lncRNAs) were identified. One specific lncRNA, lncRNA16397, was shown to function as an anti-sense transcript or to regulate the expression of *SlGRX22*. Additionally, overexpression of *lncRNA16397* triggered increased expression of *SlGRX21* [94]. *S. lycopersicum* that overexpressed *lncRNA16397* and *SpGRX* exhibited fewer disease symptoms and lower reactive oxygen species (ROS) accumulation compared to wild type plants when infected with *P. infestans*. These findings suggest that *lncRNA16397* enhances *SlGRX* expression, which in turn reduces ROS buildup and mitigates cell membrane damage, ultimately improving resistance to *P. infestans* [95]. A comparative analysis of lncRNA expression in resistant *Gossypium barbadensecv* was conducted to explore their potential roles. The functional analysis revealed that silencing two key lncRNAs, GhlncNAT-ANX2- and GhlncNAT-RLP7, led to increased resistance in seedlings against *Verticillium dahliae* and *Botrytis cinerea*. This enhanced resistance may be linked to the increased expression of *LOX1* and *LOX2* [93].

By sequencing the susceptible *S. lycopersicum* line JS-CT-9210 under both TYLCV-infected and -uninfected conditions, researchers identified 1767 lincRNAs and 289 NAT-lncRNAs. Further analysis using virus-induced gene silencing (VIGS) revealed that suppressing the expression of lncRNA Sslylnc0957 enhanced resistance to TYLCV in susceptible *S. lycopersicum* plants [8]. A model involving non-coding RNAs was proposed to investigate the molecular interactions between TYLCV and tomatoes as well as the mechanisms underlying disease progression. The reduced expression of lncRNA SlLNR1 was linked to stunted growth and curled leaves, which resemble the symptoms of TYLCV symptoms. These findings suggest that this lncRNA may interact with virus-derived small RNAs to regulate disease development during TYLCV infection [96]. A study identified 567 lncRNAs in *O. sativa* leaves infected with *Xanthomonas oryzae*, with many of their targets meaningfully associated with the JA pathway. Co-expression analysis demonstrated that 39 protein-coding genes associated with JA interacted with 73 lncRNAs. This suggests that these lncRNAs may have a regulatory function within the JA signaling pathway [97].

## 4. Conclusions and Perspectives

Non-coding RNA has a clear physiological function but it does not encode any protein and only acts as an RNA molecule. From the proportion of ncRNA in all transcripts of different biological cells, we can see that the quantity and diversity of RNA seem to be closely related to the complexity of species. Its universality and importance are far beyond people’s imagination, exerting a key influence on many biological processes in living organisms [98]. With the discovery of numerous non-coding genes, more and more research has involved plant long non-coding RNA. Nevertheless, studies on IncRNAs in plants are still in the early stages of development.

There are still many problems to be faced: (1) The definition of lncRNA is still controversial [93]. At present, it still depends on whether it has the structural feature of an open reading frame. Research has shown that certain lncRNAs possess ORFs that encode sequences similar to those of proteins produced by various known genes. Additionally, the method of dividing lncRNA into two parts can be questioned. LncRNA is typically characterized as having a length exceeding 200 nt; however, this criterion is somewhat subjective since numerous non-coding RNAs that are shorter than 200 nt do not belong to either small RNA or structural RNA categories. Therefore, the categorization scheme for lncRNA is not yet fully understood [99]. (2) Beginners often find it challenging to grasp lncRNA information quickly, and opportunities for cross-referencing are limited, making it difficult to clarify the biological functions of lncRNA [100]. (3) A consistent naming convention for lncRNA is lacking; this makes it challenging to differentiate between functional and nonfunctional non-coding transcripts. Additionally, the variety of lncRNA categories and their functional implications mean that findings from different studies often have limited relevance to one another [21]. (4) It is difficult to identify lncRNA. Technologically, because the constituent unit of lncRNA is a nucleotide, changes in some RNAs are not sufficient to alter its main function so it is very difficult to identify lncRNA using traditional genetic methods. (5) Most of the techniques used in lncRNA research are high-throughput methods. Avoiding transcriptional interference is a great challenge, and after a large amount of lncRNA information is obtained, it is difficult to effectively analyze the structure and function [101]. The main reason is that the number of relevant databases is still very few, and the related algorithms lack development due to the lack of knowledge of lncRNA information. Therefore, it is necessary to establish more effective methods to systematically study the structure and function of lncRNA.

At present, reports about lncRNAs involved in plant stress resistance and growth and development are mainly focused on mechanistic research, with few reports in breeding. Many lncRNAs have been identified and research has progressed on their molecular mechanisms in plant proliferation, progression, and stress responses, suggesting that lncRNAs could significantly impact agricultural breeding and the improvement of germplasm. LncRNAs exhibit considerable diversity and can influence gene expression through epigenetic, transcriptional, and post-transcriptional mechanisms. They are involved in various functions related to progression and growth, stress resilience, physiological processes, and disease conditions [11,102,103]. Currently, preliminary research on plant lncRNAs mainly focuses on the identification and regulatory mechanisms of lncRNAs in species, including *Arabidopsis*, *Z. mays*, *T. aestivum*, and *O. sativa*. Additionally, researchers are investigating their contributions in governing plant expansion and progression, stress responses, reproductive processes, and defense against pathogen attacks [104,105,106]. Transcriptomics research has shown that many lncRNAs are induced by stress conditions. It is likely that some of these induced lncRNAs are cryptic transcripts arising from the over-expression of transcription factors and the presence of accessible chromatin. A remarkable challenge in this area is determining which lncRNAs genuinely function as regulatory elements. A comprehensive insertion mutagenesis initiative aims to discover *Arabidopsis* lines with T-DNA insertions in the non-coding regions of the genome. Additionally, large-scale RNAi to knockdown lncRNAs combined with phenotypic characterization will aid in finding unique lncRNAs that undertake imperative responsibilities in the plant life cycle. The effectiveness of T-DNA insertion mutants for investigating SVK (uns-1) and HID1, along with the application of RNAi for examining APOLO, is clearly established. Genome editing techniques that involve RNA binding can contribute significantly to the research of lncRNAs. Additionally, manipulating lncRNAs at their native genomic location through these tools presents a valuable approach for exploring their functions [9].

In some instances, the molecular processes underlying the function of lncRNAs have been clarified. Specifically, APOLO, COLDAIR, and COLDWRAP perform duties in the development of chromatin loops. In these cases, the chromatin loops function as inhibitors, although their precise task remains unclear. Multiple scenarios exist, including the possibility that the loop alone is enough to suppress gene expression or that it facilitates the recruitment of repressive proteins to the locus. For APOLO and COOLAIR, another mechanism of action involves the generation of R-loops. A comprehensive investigation of R-loop across the *Arabidopsis* genome has been performed. The subsequent challenge is to identify lncRNAs linked to this process, which will contribute to advancing the field. For DOG1-asDOG1, HSF2a-asHSFB2a, and FLO-RECDF5, mutual repression through sense–anti-sense interactions has been demonstrated, indicating that RdDM does not appear to take part in this repression [107].

The mechanism by which this repression is achieved is an intriguing open question. Possibilities such as RNA polymerase collisions, sequestration, and chromatin modifications are worth exploring. Similar to other model organisms, various lncRNAs in *Arabidopsis* are known to bind and attract different proteins—including chromatin modifiers—to specific target sites or sequester proteins to hinder their activity. LHP1 interacts with APOLO, while CLF associates with COOLAIR and COLDAIR. LncRNAs produced by Pol V within the RdDM pathway attract DNA methyltransferases and histone-modifying enzymes to their target sites. While both LHP1 and CLF have the ability to bind RNA, they do not possess RNA recognition motifs. Instead, the amino acid residues responsible for RNA binding are scattered throughout the proteins [108]. This prompts an inquiry into how lncRNAs selectively bind to regulatory sites in the absence of RNA recognition motifs. It is a complex challenge to experimentally identify a universal RNA binding mechanism in proteins that lack these motifs [109]. Interactions between lncRNAs and proteins are infrequently observed in *Arabidopsis*. However, by utilizing and enhancing techniques for investigating RNA–protein interactions, researchers could gain further insights into the molecular mechanisms underlying lncRNA function. They also employed computational and experimental methods to identify secondary structures in lncRNAs and the RNA-binding properties of chromatin modifiers [110,111].

This review highlights the essential functions of lncRNAs in plant expansion and progression as well as their roles in response to biotic and abiotic stresses. A single gene can be influenced by various ncRNAs and lncRNAs may operate in multiple ways rather than functioning in isolation. In contrast, lncRNAs can engage with numerous genes and proteins and their mechanisms are intricate. Conspicuously, the structure, function, and origin of lncRNAs in both animals and plants show significant similarities, adhering to specific principles. As technologies like gene chips and high-throughput sequencing advance, an increasing number of plant lncRNAs will be identified and analyzed in greater detail. This advancement is crucial for elucidating the mechanisms of non-coding RNAs and enhancing their potential applications. It allows us to gain a more comprehensive understanding of the functions and regulatory mechanisms of plant non-coding RNAs, thereby enriching our knowledge of the essence of plant life activities. Research on plant non-coding RNAs not only helps to uncover the mysteries of plant genetic regulation but also provides new ideas and methods for breeding stress-resistant, high-yielding, and high-quality crop varieties. As investigations into plant non-coding RNAs deepen, a large amount of relevant data are accumulated. Reviewing these developments promotes the creation of bioinformatics tools, such as non-coding RNA databases, prediction models, and analytical software, which improves our capacity to identify and predict the functions of plant non-coding RNAs. It also helps to promote interdisciplinary collaboration, integrate research results from different fields, and drive the study of plant non-coding RNAs to a deeper level. Reviewing the progress in the study of plant non-coding RNAs allows us to pinpoint the shortcomings and challenges in current research, offering guidance and ideas for future investigations. This could involve conducting a more thorough exploration of certain types or functions of non-coding RNAs that remain underexplored.

## Figures and Tables

**Table 1:** Classification and functional archetypes of lncRNA.

Classification	LncRNA Type	References
lncRNA function	Decoy archetype	[25,26,27]
	Scaffold archetype	
	Guide archetype	
	Signal archetype	
Position on genome	Enhancer lncRNA	[28,29]
	Co-transcriptional lncRNA Anti-sense lncRNA	
	Intronic transcript lncRNA	
	Large intergenic ncRNA	

**Table 2:** The databases of plant lncRNAs.

Database	Website	References
CANTATA	http://yeti.amu.edu.pl/CANTATA/ accessed on 12 September 2024	[54,55]
NONCODE	http://www.noncode.org/ accessed on 12 September 2024	[56]
GreeNC	http://greenc.sequentiabiotech.com/wiki2/Main_Page accessed on 5 November 2024	[57]
PLncDB	https://bis.zju.edu.cn/PlncRNADB/index.php accessed on 21 September 2024	[58]
JustRNA	http://JustRNA.itps.ncku.edu.tw accessed on 19 October 2024	[16]

## Data Availability

The data presented in this study are available upon request from the corresponding author.
